# Peripheral and Thymic Foxp3^+^ Regulatory T Cells in Search of Origin, Distinction, and Function

**DOI:** 10.3389/fimmu.2013.00253

**Published:** 2013-08-27

**Authors:** Chetan Dhamne, Yeonseok Chung, Amin Majid Alousi, Laurence J. N. Cooper, Dat Quoc Tran

**Affiliations:** ^1^Department of Paediatrics, University Children’s Medical Institute, National University Hospital, Singapore; ^2^Institute of Molecular Medicine, Center for Immunology and Autoimmune Diseases, UTHealth, Houston, TX, USA; ^3^Department of Pediatrics Patient Care, Division of Pediatrics, M.D. Anderson Cancer Center, Houston, TX, USA; ^4^Department of Stem Cell Transplant and Cellular Therapy, Division of Cancer Medicine, M.D. Anderson Cancer Center, Houston, TX, USA; ^5^Department of Pediatrics, Divisions of Allergy/Immunology, Pediatric Research Center, UTHealth, Houston, TX, USA

**Keywords:** Foxp3, regulatory T cells, Tregs, immunological tolerance, autoimmunity

## Abstract

Over the past decade, much has been learnt and much more to discover about Foxp3^+^ regulatory T cells (Tregs). Initially, it was thought that Tregs were a unique entity that originates in the thymus. It is now recognized that there is a fraternal twin sibling that is generated in the periphery. The difficulty is in the distinction between these two subsets. The ability to detect, monitor, and analyze these two subsets in health and disease will provide invaluable insights into their functions and purposes. The plasticity and mechanisms of action can be unique and not overlapping within these subsets. Therefore, the therapeutic targeting of a particular subset of Tregs might be more efficacious. In the past couple of years, a vast amount of data have provided a better understanding of the cellular and molecular components essential for their development and stability. Many studies are implicating their preferential involvement in certain diseases and immunologic tolerance. However, it remains controversial as to whether any phenotypic markers have been identified that can differentiate thymic versus peripheral Tregs. This review will address the validity and controversy regarding Helios, Lap/Garp and Neuropilin-1 as markers of thymic Tregs. It also will discuss updated information on distinguishing features of these two subsets and their critical roles in maternal-fetal tolerance and transplantation.

## Introduction

Since the identification of regulatory T cells based on CD25 expression by Sakaguchi et al. there has been a quest to decipher their mechanisms of suppression, to identify their functional role in different diseases and to develop therapeutic strategies to cure disorders of immune dysregulation (1, 2). Subsequently, the transcription factor, Foxp3, was discovered as a critical lineage molecule necessary for their development and function (3–5). This discovery fortified the pillar that established their true existence and set in motion a wide spread investigation of their role in health and disease. Many suppressor functions of Tregs have been described, although we have not been able to identify one central mechanism of action (6). With the revelation that naïve CD4^+^ T cells can be differentiated to become Foxp3^+^ T cells, we now appreciate that a Treg population can constitute various subsets, particular those derived from the thymus and the periphery. There have been excellent reviews on distinguishing features of these subsets (7–11). With the discovery and better characterization of these subsets, the nomenclature is becoming more variable and often times confusing. It is difficult to know whether induced Tregs (iTregs) is referring to those generated *in vitro* or *in vivo*. Similarly, the term natural Tregs (nTregs) is often used for Tregs in the peripheral blood of humans or lymphoid organs of animals with the assumption that they had originated from the thymus, when in fact they can be a composition of thymic and peripheral derived Tregs. In this review, these terms will be used to refer to a specific subset of Tregs: (1) Tregs = all subsets, (2) tTregs = thymic derived, (3) pTregs = *in vivo* peripheral derived, and (4) iTregs = *in vitro* iTregs. A recent recommendation to simplify the nomenclature has been proposed (12). However, we feel that the elimination of subscript and the word “cell” would make it more simplistic and less verbose.

Several studies have shown that certain mouse strains thymectomized at or before 3 days after birth led to autoimmune damage of various organs like thyroid, stomach, ovaries, and testes and the appearance of tissue-specific autoantibodies in the circulation (13, 14). It is possible that tTregs are involved in controlling tissue-specific autoimmunity. It has been challenging to study the *in vivo* development of pTregs because of a lack of biomarkers to identify them. Rudensky’s group recently investigated the role of three conserved non-coding DNA sequence (CNS) elements at the Foxp3 locus in regulating Treg development (15). They revealed that CNS1, which possesses a TGFβ-NFAT response element, has a dominant function in pTreg differentiation in gut-associated lymphoid tissues. Subsequently, they demonstrated that selective blockade in differentiation of pTregs in CNS1^−/−^ mice did not lead to unprovoked multi-organ autoimmunity, exacerbation of induced tissue-specific autoimmune pathology or increased proinflammatory responses to Th1 or Th17 cells (16). However these mice spontaneously developed remarkable Th2 type pathologies at mucosal sites in the GI tract and the lungs with hallmarks of allergic inflammation and asthma. Furthermore, they had altered gut microbiota, suggesting the important involvement of pTregs in regulating intestinal immunity and microbes. These studies indicate that tTregs are sentinels of systemic and tissue-specific autoimmunity, while pTregs serve a distinct and essential function in controlling adaptive immunity to restrain allergic type inflammation at mucosal surfaces. In response to inflammation and integration of environmental cues, Tregs can function to limit collateral damage (17). After eradication of the invading pathogens, the induction of pTregs can serve as peacekeepers to suppress antigen specific response and prevent emergence of cross-reactive T cells. Accordingly failure of these mechanisms can result in immune mediated diseases.

A few trials with Treg immunotherapy have shown promising results, but clinical translation has been difficult because of our inability to fully characterize these cells and understand their mechanism of action and factors that maintain their stability in the face of immune activation. We now recognize that there are varieties of regulatory T cells based on their origin of development (7). There are unique subsets of cells that contribute to the regulatory function like IL10 producing Tr1 cells, TGFβ producing Th3 cells, CD8^+^ Tregs, natural killer (NK) regulatory T cells, and regulatory B cells (Bregs). Thus the immunosuppressive cells are more complex than we had thought earlier. These discoveries open up new frontiers to understand the role of these distinct subsets of immunosuppressive cells in different situations. In this special issue, we will restrict our focus on the different subsets of Foxp3^+^ Tregs as indicated in Table 1. We will provide an updated knowledge and issues regarding whether these markers are truly tTreg specific: Helios, latency associated peptide (Lap)/Garp, and Neuropilin-1. We will highlight our current understanding of differences in generation, maintenance, survival, and function of these Treg subsets. Accurately distinguishing pTregs from tTregs will help to clarify the biological features and contributions of each subset in maternal-fetal tolerance transplantation. Finally we will touch briefly upon the challenges we face in adoptive transfer of these cells from bench to bedside. Table 1 provides a summary of some of the distinguishing features of the different Treg subsets.

**Table 1:** **Distinguishing features of Treg subsets**.

	tTregs	pTregs	iTregs
Origin	Thymus	Periphery	*In vitro*
Growth/development requirement	Cytokine: IL2 (68, 69)	Cytokines: IL2, TGFβ (74, 75, 98)	Cytokines: IL2, TGFβ (74, 75, 98)
	Costimulation: CD27 (81), CD28 (70–73), CD40L (80)	Costimulation: TLR2 (?) (66)	Costimulation: CD28 (76)
	Epigenetics: CNS3 (15)	Modulators: retinoic acid (78, 79)	Modulators: retinoic acid (79)
		Epigenetics: CNS1 (15)	
Biomarkers	Low TSDR methylation (86)	Intermediate TSDR methylation (?) (23, 24)	Intermediate TSDR methylation (87)
	Helios+ (?) (21, 23, 24)	Helios− (?)	
	Neuropilin-1+ (?) (44, 45)	Neuropilin-1− (?)	
	LAP+ (?) (38)	LAP− (?)	
	LRRC32/GARP+ (?) (36)	LRRC32/GARP− (?)	
Antigen recognition	High-affinity TCR Predominantly self-antigens (53–60)	Chronic/suboptimal TCR stimulation (59, 61, 62)	
		Environmental/microbial antigens (65–67)	

*tTregs = thymic derived, pTregs = in vivo peripheral derived, and iTregs = in vitro induced Tregs. Numbers in parenthesis are references. Question mark indicates controversial or unknown*.

## Phenotypic Markers

### Helios

Ever since the recognition that Tregs can be generated in the peripheral, there has been a focus in identifying phenotypic markers that can distinguish them from the tTregs. The ability to discriminate the two subsets would allow for a better understanding of their specific functions in certain diseases and immune responses. This critical information would provide for more strategic treatments and therapeutic development. Multiple reports have indicated that Tregs have the potential to be plastic and can become cytokine producers (18–20). However, in those studies, they have assumed that the Tregs (CD4^+^Foxp3^+^) obtained directly from human peripheral blood or mice were tTregs when in fact they could be a composition of tTregs and pTregs. In the absence of segregating the two subsets, it is unclear whether the plasticity is predominately from tTregs. A similar issue occurred when many of those same studies investigated the stability of Foxp3 in Tregs by utilizing elegant transgenic mice where they could track a cell that had previously expressed Foxp3. Because of this problem, we and others have been driven to search for markers that can differentiate these two subsets. We have demonstrated that Tregs from mice and humans can be subdivided into two populations based on their expression of Helios, a zinc finger transcription factor (21). Approximately 70% of Tregs in peripheral blood of humans and in peripheral lymphoid tissues of mice are Helios^+^. Over 95% of tTregs in the thymus of mice are Helios^+^. Interestingly, the vast majority of IL2^+^, IL17^+^, and IFNγ^+^ Tregs are localized within the Helios^−^ population. We and others have analyzed human cord blood and thymus specimens and have found that>90% of Tregs are Helios^+^ (22). While we cannot definitively rule out that the<10% Helios^−^ Tregs are thymic derived, they might have been generated in the peripheral during the fetal gestation for the cord blood or peripherally recirculated for the thymus. Therefore, from our study, we have concluded that Helios is a marker of tTregs and the Helios^−^ subset represents pTregs. Subsequently, we and McClymont et al. have demonstrated that the human Foxp3^+^Helios^+^ Tregs contain<10% CpG methylation in the Treg-specific demethylation region (TSDR) of the Foxp3 promoter, while the Foxp3^+^Helios^−^ subset are>40% methylated (23, 24). In addition, McClymont et al. have shown that the IFNγ^+^ Tregs from patients with type 1 diabetes are Helios^−^ and predominately methylated at the TSDR.

Since our initial report, subsequent studies from other groups have challenged our claim that Helios is a marker of tTregs. The first study showed in murine experiments using 5C.C7 Rag2^−/−^ transgenic mice that Helios could be expressed *in vitro* in iTregs and *in vivo* in pTregs (25). Their findings revealed that *in vitro* expression of Helios in iTregs was dependent on the presence of antigen-presenting cells (APCs). Using a 5C.C7 Rag2^−/−^ CD45.1^+^ T cells adoptively transferred into B10. A wild-type recipients and low dose immunization with intravenous injection of moth cytochrome c (MCC) peptide, they were able to observe induction of pTregs with the majority expressing Helios. Since these were transgenic mice that lack tTregs, similar experiments using polyclonal CD4^+^Foxp3-GFP^−^ cells from wild-type mice would be necessary to confirm that the expression of Helios could be induced in these cells. Another study using human experiments presented data indicating that tTregs could be Helios^−^ (26). They based this claim from the observation of a few healthy donors (age unknown) that ∼30–40% of naïve Foxp3^+^ Tregs in the peripheral blood were found to be Helios^−^, regardless of the combinations of naïve markers used: CD45RA, CCR7, CD62L, and CD31. The challenge with human system is that things are in a dynamic state, particularly the peripheral blood which is a highway for trafficking from one site to another. Therefore, it is unclear whether the “naïve” Helios^−^ Tregs had been stimulated recently to become pTregs but had not yet altered their naïve markers. There is no evidence to support that the down-regulation of these markers is a permanent state as the naïve cell becomes a memory cell. Since>90% of cord blood Tregs are Helios^+^, some of these naïve tTregs can lose their expression of Helios over the human lifespan. If this process is true, then the absence of Helios expression is not a stringent marker for pTregs. Contrary to our results, their study showed that sorted human CD4^+^CD45RA^+^Foxp3^+^Helios^+^ and Helios^−^ Tregs have similar low methylation profile in the TSDR. Based on all the current data thus far, it is evident that Tregs can be subdivided into two subsets based on Helios expression. However, at this point it is controversial whether Helios accurately defines tTregs. A recent discovery by Rudensky et al. shed new light into the role of conserved non-coding DNA sequence (CNS) elements in the Foxp3 locus for determining the fate of tTregs and pTregs (15). Their study indicates that CNS3 is essential for the development of tTregs and pTregs. While CNS1, which contains the TGFβ-NFAT response element, plays a major impact in pTregs generated in gut-associated lymphoid tissues, it is not absolutely obligatory. CNS1^−/−^Foxp3-GFP^−^ T cells still possessed the capacity to convert into pTregs *in vivo*, although significantly less than wild-type controls. Unfortunately, there is no mention of whether the Tregs in CNS1^−/−^ mice are predominately Helios^+^. Therefore, in the absence of a lineage marker that can truly identify pTregs, we are left with correlative markers that might be influenced by different diseases and microenvironment.

Our work has ignited an intense investigation into these two subsets. Several studies have examined whether there is a preferential expansion or selection of either subset in human diseases. Elkord et al. observed that there was an increased frequency of Helios^+^ Tregs in the peripheral blood of patients with renal cell carcinoma, particularly after IL2 treatment (27). Another study revealed that there was a selective preservation of the Helios^+^ Tregs in kidney transplant recipients that received thymoglobulin induction and a reduction in control patients (28). Similarly, others have noted an expansion of Helios^+^ Tregs in patients with active systemic lupus erythematosus (29). In a murine model of human glioblastoma multiforme, the study demonstrated that the tumor-infiltrating Tregs were of thymic origin based on their expression of Helios and reduction after thymectomy (30). A recent study utilized an *in vitro* stimulation assay with T cells and monocytes to identify that the proliferation of Helios^+^ Tregs was inhibited by IL12 produced from CD16^+^ monocytes, while the Helios^−^ Tregs were inhibited by TNFα from CD16^−^ monocytes (31). In our initial study, we were unable to identify the function of Helios in Tregs. However, a recent study has indicated that Helios can regulate IL2 production in Tregs by inducing epigenetic silencing of IL2 gene expression (32). This finding does support our observation that the vast majority of IL2 production in Foxp3^+^ Tregs is localized within the Helios^−^ subset. At this point, more studies are needed to acquire a better understanding of the role of these two subsets in human diseases and whether they are distinct entities or alter egos.

### Lap/Garp complex

Although Lap, a component of latent TGFβ, was found to be expressed on the surface of Tregs, it was unclear how this pleiotropic cytokine was attached to the membrane (33, 34). Another study has identified Garp (Lrrc32) as a Treg-specific cell surface molecule that has suppressive function and the ability to induce Foxp3 expression (35). However, it was unknown how Garp mediated these functions. Recently, we and others have demonstrated that Garp (Lrrc32) is the membrane anchoring molecule that binds to latent TGFβ within the Tregs and facilitates its surface expression (36, 37). Therefore, surface Lap on Tregs is a complex of Garp, Lap, and active TGFβ. We and others have shown that surface Garp and Lap expression selectively identifies activated Tregs that represent a stable subset with highly potent suppressive function (38, 39). The vast majority of cytokine-producing Foxp3^+^ Tregs are within the Lap^−^ subset. Moreover, the iTregs fail to express surface Lap or Garp. Based on these observations, we have established that the selection of Lap^+^ Tregs is an efficiency method to repurify bona fide Tregs from the contaminating Lap^−^ Tregs and Foxp3^−^ T cells during Treg expansion. We believe that the Lap^+^ Tregs represent a highly potent and stable subset ideal for Treg immunotherapy. However, it remains controversial as to whether this membrane-bound TGFβ is involved in the development, maintenance, or suppressor function of Tregs (40). A recent study showed that Garp-transgenic mice with forced expression of Garp on all T cells resulted in reduction of Tregs in the thymus and periphery (41). A subsequent study observed that transgenic mice with Garp-deficient Tregs developed normally (42). The absence of Garp on the Tregs did not compromise their suppressive function. Instead, the membrane-bound TGFβ was important for induction of both Th17 and pTregs/iTregs. Along the same line, we and others have recognized that IL1 receptors are preferentially expressed on activated Tregs but not on iTregs (38, 43). While the receptors (CD121a and CD121b) do not appear to be involved in Treg suppressor function, they might play an important role in regulating the development of Th17 and pTregs. Nonetheless, it remains unclear whether Lap^+^ or CD121a^+^/CD121b^+^ Tregs are derived from the thymus. Interestingly, the study by Shevach et al. has demonstrated that mouse iTregs and pTregs could express Garp, which is contradictory to our human studies (42).

### Neuropilin-1

There have been several claims that neuropilin-1 (CD304) is a surface marker of Tregs (44, 45). It can function to enhance the interaction between Tregs and dendritic cells (DCs) during antigen recognition (46). Another possible function of CD304 is for mediating Treg infiltration into the tumor microenvironment (47). In this study, the authors showed that mice with specific deletion of CD304 in T cells were less susceptible to tumor growth. However, adoptive transfer of WT Tregs in these mice significantly increased the tumor growth, suggesting the role of CD304 in mediating Treg migration into the tumor site to modulate anti-tumor immune responses. Recently, two studies demonstrated in murine models that CD304 can distinguish tTregs from iTregs and pTregs (48, 49). The first study used myelin basic protein (MBP)-specific TCR transgenic mice (1B3) crossed with Rag^−/−^ mice to show that pTregs could spontaneously develop after 3 weeks in these mice, but interestingly the pTregs had absence to low CD304 expression unlike the Tregs from WT controls (48). Even with the generation of pTregs, these mice still developed experimental autoimmune encephalomyelitis (EAE) by 3–4 weeks of age. Moreover, in an EAE model, the adoptive transfer of these pTregs failed to attenuate the disease as compared to total Tregs or CD304^hi^ Tregs. The second study also revealed similar evidence for the differential expression of CD304 on tTregs versus pTregs, except that in the inflamed tissues such as the spinal cords from EAE or the lungs from OVA-induced asthma mice, a large portion of the pTregs were found to express high level of CD304 (49). While these murine studies are insightful to our understanding of Treg development and potential biomarkers, the translation into human studies can be controversial. We have not been able to appreciate much expression of CD304 on human Tregs in peripheral blood of healthy donors and in Tregs during *in vitro* expansion (50). Another study also argues against the applicability of CD304 as a marker of human Tregs (51). That study showed that CD304 was not differentially expressed on human Tregs from thymus, blood, lymph nodes, and tonsils. Similarly, a different study also exposed that CD304 was not a selective marker of human Tregs in lymph nodes or peripheral blood (52). Therefore, the data do not support CD304 as a marker of human tTregs. However, Tregs expressing CD304 represent a unique subset of Tregs that appear to possess distinguished properties and functions.

Overall, there is a discrepancy between the mouse and human studies regarding Helios, Garp, and CD304 as markers that can differentiate tTregs from the other subsets of Tregs. The evidence thus far would indicate that murine data are not translatable to human and therefore should be interpreted with caution. Human studies should continue to investigate these subsets of Tregs to gain more insights into their functions and roles in different diseases and inflammatory conditions. At this point, we still lack a definitive lineage biomarker to identify between tTregs and pTregs.

## Distinguishing Features

### Antigen specificity and affinity

tTregs are generated in the thymus by positive selection when MHC class II restricted self-peptides with high-affinity are presented to CD4^+^ thymocytes (53–55). The thymic medulla appears to be the critical compartment for their development (56). Their signal strength of TCR stimulation is greater than that required for positive selection and lower than that required for negative selection. In MHC class II restricted transgenic TCRs expressed in a Rag2^−/−^ mice, positive selection resulted in development of CD4^+^ thymocytes but not tTreg cells (57). On the other hand, a low affinity antigen would result in the generation of fewer CD4^+^CD25^+^ cells (58–60). Therefore, signal strength plays an important role in directing CD4^+^ thymocytes in the thymic medulla toward tTreg lineage.

pTregs are generated in the periphery from naïve CD4^+^CD25^−^ T cells preferentially in the peripheral lymphoid tissues. Elegant experiments by Apostolou et al. and Thorstenson et al. showed CD4^+^CD25^−^ T cells from Rag^−/−^ TCR transgenic mice adoptively transferred into antigen-expressing transgenic mice or mice that have received intravenous or oral tolerizing dose of peptide antigen can be converted to a CD4^+^CD25^+^ regulatory T cells (59, 61). Gottschalk et al. have shown that a low antigen dose of a high-affinity TCR ligand is optimal to induce a persistent population of pTregs *in vivo* (62). Similarly, high doses of peptides or polyclonal TCR stimuli could prevent Foxp3 induction via NFκB-dependent cytokine production (63, 64). Therefore tTregs are generated in the thymus in response to intermediate/high-affinity interaction with self-antigen; whereas pTregs are induced in the periphery in response to a low/suboptimal dose of high-affinity alloantigen. Another source of antigens for peripheral education of pTregs could come from colonic commensal microbiota (65–67). Intestinal microbiota such as *Clostridium* species can promote induction of colonic pTregs that correlates with increased bioavailability of TGFβ (67). In the Lathrop et al. study, the colonic Tregs have a different TCR repertoire than Tregs from other peripheral sites (65). These unique TCRs are not involved in tTreg development. In the Round and Mazmanian study, they revealed that polysaccharide A from *Bacteroides fragilis* can mediate the generation of IL10 producing pTregs via Toll-like receptor 2 (TLR2) signaling (66). It appears that the generation of pTregs is more complex than simply TCR signaling alone. A collaboration of other signaling pathways such as TGFβ, IL2, retinoic acid, TLRs, and cytokine milieu are needed to direct a naïve T cell toward a pTreg or other effector subsets.

### Costimulation

Interleukin-2 (IL2) and strong CD28 costimulation are essential for the development of tTregs. Knockout mice of IL2R^−/−^ and CD28^−/−^ failed to generate tTregs and developed severe lethal autoimmunity early in life (68, 69). IL2 is important but might not be necessary for tTreg development and CD28 stimulation may be the most important factor for their development (70–72). In contrary, a recent study has created Treg-specific CD28 conditional knockout mice and interestingly, they have normal numbers of tTregs (73). However, these mice developed severe autoimmunity due to profound proliferative and survival dysfunction in the Tregs. TGFβ, though not involved in driving tTreg development and lineage commitment, might provide useful signals for survival during early tTreg development (74). On the other hand, IL2 and TGFβ are required for generation of iTregs (75, 76) While CD28 signaling appears to be important for iTreg generation (77), strong CD28 costimulation is detrimental by mediating downstream lymphocyte-specific protein tyrosine kinase (Lck) signaling (78, 79). Molecules that can modulate the CD28 costimulation would influence the differentiation of pTregs, such as the case for all-trans retinoic acid. In this study, the treatment with all-trans retinoic acid during *in vitro* culture of naïve T cells with DCs expressing high level of CD80/CD86 costimulatory molecules resulted in enhanced induction of iTregs (80). One possible explanation is that all-trans retinoic acid can increase histone methylation and acetylation within the promoter and CNS elements at the Foxp3 gene locus (81).

Ultimately, it is the APCs that are the key regulators of Treg development. It has been suggested that plasmacytoid DCs in the human thymus could promote the development of CD4^+^CD25^+^Foxp3^+^ tTregs when activated with CD40 ligand (CD40L) and IL3 (82). Recently, a new study has revealed that CD27-CD70 costimulatory pathway is essential for tTreg development by rescuing them from apoptosis, subsequent to Foxp3 induction by TCR and CD28 signals (83). The CD70 on medullary thymic epithelial cells (mTECs) and DCs in the thymic medulla triggers the CD27 signal on tTregs to promote their survival by inhibiting the mitochondrial apoptosis pathway. In contrast, CD103^+^ DCs that are found in the mesenteric lymph nodes and lamina propria of the small intestine can enhance the conversion of pTregs (84, 85). In peripheral lymphoid tissue, CD8^+^CD205^+^ splenic DCs appear to play a specialized role in pTreg development by producing TGFβ (86). Thus the APCs, the microenvironment, cytokine milieu, and costimulatory molecules all collaborate in the generation and maintenance of tTregs and pTregs.

### Stability and plasticity

tTregs appear to be more stable *in vivo* probably due to the continuous exposure to self-antigens. IL2 and TGFβ are required for Treg stability and regulatory function. While TGFβ1 is not required for thymic development of Tregs, it is essential for the maintenance of Foxp3 expression, suppressor function, and survival in the periphery (87). This phenomenon is likely due to the methylation status at the Foxp3 TSDR region. tTregs show consistently demethylated TSDR region and are a more stable pool of suppressive cells in the presence of IL2 (88). The level of TSDR demethylation can discriminate Tregs from *in vitro* iTregs or activated Foxp3^+^ conventional T cells (89). In the presence of inflammatory cytokines like IL6, Tregs lose their Foxp3 expression, are less suppressive and a certain percentage of them convert to pathogenic memory T cells (90, 91). A potential issue with these studies is that they assume the Tregs are tTregs instead of a composition of tTregs and pTregs. It is possible that the instability is coming from the pTreg subset. In support of this notion, a subsequent study refuted this debatable topic of Treg plasticity by demonstrating the stability of Tregs under physiologic and inflammatory conditions (92). This study also uses genetic fate mapping technical to track Tregs, even after they had lost Foxp3 expression. Unlike continuous labeling used in previous studies, this study utilizes inducible labeling of Foxp3 expressing cells to eliminate the constant incorporation into the labeled cells that had transiently up-regulated Foxp3. This strategy enables accurate assessment of bona fide Treg maintenance and stability. There is still considerable debate on this topic that needs to be resolved because of its important implications in diseases and therapeutic applications (93).

The question of whether iTregs are stable and can be manufactured in human continues to be of great interest, because the ability to create Tregs with different antigen and homing specificities offers enormous therapeutic potentials. The human iTregs generated from naïve T cells are not anergic, non-suppressive, transient, and highly methylated in TSDR (89, 94, 95). It appears that Foxp3 is promiscuous and has other novel functions in conventional T cells (96). One possible explanation for the lack of regulatory phenotype in human iTregs is their inability to achieve high and sustained level of Foxp3 expression. Lentiviral-based overexpression of Foxp3 can reprogram naïve and memory CD4^+^ T cells to possess similar phenotype and function as *ex vivo* Tregs (97). Several studies have suggested that iTregs are stable *in vivo*, even under inflammatory conditions (98, 99). However, other studies have revealed that iTregs and pTregs are highly unstable under certain conditions. iTregs depend on IL2 and STAT5 signaling *in vivo* to stabilize their Foxp3 expression (100). Suppressor of cytokine signaling 2 (SOCS2) protein is equally important to prevent IL4 induced Foxp3 instability and secretion of proinflammatory cytokines in iTregs and pTregs (101). Signaling through receptors for C3a and C5a can also negatively impact the generation, function, and stability of iTregs and pTregs (102). Of most concern from a therapeutic standpoint is the possibility of reversion into pathologic, non-Tregs, as demonstrated in a murine study showing that alloantigen-specific iTregs can rapidly revert *in vivo* and fail to protect experimental graft versus host disease (GVHD) (103). While this finding is controversial, it still raises a concern that needs to be monitored and approached with caution in human clinical trials.

## Disease Association

### Tregs in maternal-fetal tolerance

Pregnancy is a physiological condition in which tolerance to paternal alloantigens is critical for coexistence of the mother and fetus across the placental barrier. Accumulating data indicate that Tregs play a pivotal function in immune tolerance during pregnancy (104, 105). During pregnancy there is an increase in the number of Tregs in pregnant mice and humans (106, 107). Antibody mediated depletion of Tregs during pregnancy led to increased reabsorption of embryos and reduced litter size in allogeneic matings in mice (108, 109). Women with decreased Treg numbers had increased rates of abortion and preeclampsia (110, 111). Treg expansion was shown to be essential for tolerance of the semi-allogeneic fetus in healthy pregnancy and was impaired in preeclampsia in humans (112). With regard to the subsets of Tregs, there was an expansion of Helios^−^ Tregs over the Helios^+^, particularly in the decidua during healthy pregnancy (113). In preeclampsia, this preferential expansion of Helios^−^ Tregs was impaired. All of these studies beg the question regarding which subset of Tregs is more critical during reproduction. To address this question, Rudensky group utilized their CNS1^−/−^ mice that have impaired development of pTregs to investigate their role in maternal-fetal immune tolerance (114). The study reported that mating CNS1^−/−^ female mice with allogeneic but not syngeneic males resulted in increased fetal resorption. There was insufficient generation of pTregs in the decidua, leading to increased immune cell infiltration and defective remodeling of spiral arteries. It remains unclear as to the source of TGFβ and the APCs involved in the induction of pTregs. One study suggests that trophoblast cells can be involved in the recruitment and induction of iTregs based on *in vitro* culture data (115). The study shows that trophoblast cell lines, Swan-71 and HTR8, constitutively secrete high levels of TGFβ for the induction of iTregs. We now have a better understanding of maternal-fetal tolerance and the importance of Tregs, particularly the pTreg subset.

### Tregs in transplantation

In hematopoietic stem cell transplantation, graft rejection or GVHD occurs when the activated CD4^+^ and CD8^+^ T cells recognize alloantigen expressed on MHC presented by self or allo APCs and initiate an immune response against self. Current methods of immunosuppression using calcineurin or mTor inhibitors or antimetabolites are clearly insufficient as rates of mortality and morbidity associated with GVHD remain high. Adoptive transfer of Tregs has shown promise in mouse models to suppress autoimmune disease, prevent graft rejection and GVHD in hematopoietic stem cell transplantation (50, 116, 117). Acute GVHD typically occurs in a relatively short window between 1 and 3 months after which central tolerance develops and provides lifelong protection against adverse allo-responses. The predictable timeline of this immune phenomenon and its potential to cause significant morbidity and mortality makes it a good indication for adoptive Treg therapy (118–120).

While murine data are very promising, there are practical problems in translating Treg therapy to the clinic. First and foremost, we have not characterized Tregs enough to isolate a pure population of human CD4^+^Foxp3^+^ Tregs. Using magnetic bead separation under cGMP conditions, we can isolate between 60 and 70% CD4^+^CD25^+^Foxp3^+^ cells with the majority of the contaminants being CD4^+^CD25^+^Foxp3^−^ cells (38, 121). Secondly we do not have sufficient numbers as 1:1 Treg to effector T cell ratio is required to get effective immunosuppression (122). Thus there is a need to expand these cells *ex vivo* to achieve sufficient numbers. But Tregs are anergic to begin with and difficult to expand. Expansion protocols using anti-CD3/CD28 conjugated beads can generate sufficient number of Tregs but the expanded cells cannot maintain their Foxp3 expression and would lose their suppressive potential. To overcome some of these hurdles, Hippen et al. have generated large numbers of Tregs for clinical use by stimulating Tregs in the presence of rapamycin with anti-CD3 antibody-loaded, cell-based artificial antigen-presenting cells (aAPCs) that expressed the high-affinity Fc receptor and CD86 (123). These cells maintained their Foxp3 expression and suppressive function when infused into humanized GVHD mouse model. Infusion of Tregs has been shown to be safe. In these trials there was no statistically significant difference in rates of relapse, graft rejection, and infections (124). In fact as shown by Di Ianni et al. immune reconstitution was faster since these patient did not receive prolonged immunosuppression using pharmacological agents (125). In the Minnesota trial using umbilical cord blood Tregs, rate of grade 3–4 GVHD was 43% as opposed to 61% in historical controls (126). GVHD suppression was best when Tregs were detected on day 14 post infusion and there was minimal or no suppression when Tregs lasted only about 3 days, indicating that the longevity of Tregs made the difference. In the Italian trial using freshly isolated Tregs, only 2 out of 28 patients developed acute GVHD, but overall survival was not superior to controls (125). Infusion of Tregs is still a concern because of their instability and potential to convert to effector T cells. Adoptively transferred Tregs can convert to Th17 cells or helper T cells especially in lymphopenic host with potential pathologic effects (90, 127, 128). The plasticity of Tregs is most susceptible in an inflammatory environment in the presence of IL6 (91, 129). The issue of stability and homogeneity of Treg therapeutic products have been a major concern for us. It should be noted that expansion of Tregs is a composition of tTregs, pTregs, and contaminating non-Tregs. At this time, it is unclear whether the detection of these reverted or unstable Tregs are coming from the pTregs or tTregs. We believe that Lap^+^ Tregs represent a more homogeneous and stable population than the bulk heterogeneous parental population that has been expanded *ex vivo* for over 3 weeks (38). Ultimately like all drug manufacturing, we should strive to achieve the highest purity and homogeneity when developing a Treg product for cellular therapy in order to achieve predictable efficacy, interpretability, and minimal side effects.

Conventional CD4^+^ T cells can be induced to express Foxp3, although their suppressive functions remain controversial (94). Hippen et al. have generated clinical grade iTregs from CD4^+^ conventional T cells in the presence of TGFβ1, IL2, and rapamycin (130). These cells were much more stable and immunosuppressive in the xenogenic GVHD model. The approach of using polyclonal iTregs appears promising, but we do not know whether they will exert their immunosuppressive effect in an antigen independent manner in the human host. We do not know whether they will revert to effector cells that may have pathogenic potential as shown by Schmitt et al. in a colitis model for inflammatory bowel disease (131). It is unclear whether treatment with DNA methyltransferases and histone deacetylases inhibitors should be incorporated into the protocol to enhance their stability. Furthermore, our knowledge is lacking on the fate of these cells after they have been infused into the human body. Nevertheless, the infusion of these cells might just be sufficient to tip the balance away from an inflammatory response and induce infectious tolerance (132). Finally we might have to co-transfer Tregs and iTregs to get the best results to control GVHD after hematopoietic stem cell transplantation. Further understanding of the Treg subsets and their interaction with DCs and the cytokine milieu might help us deliver a better product for adoptive transfer.

## Discussion

A great deal of work has been accomplished in the past decade on Tregs, because of their central role in immune homeostasis, maintenance of tolerance, and regulation of inflammation. Within the Foxp3^+^ Tregs, we now appreciate that they are composed of two distinct subsets originating from either the thymus or the periphery. Murine studies indicate that CNS1 is an essential factor in the development of pTregs. These findings need to be translated in human studies to assess whether mutations in this region are associated with particular diseases. Although there are plasticity and concerns for stability in these Tregs, it appears that the pTregs are most vulnerable. Physiologically, this plasticity in the pTregs might play an important function in their diversity depending on their environment. While studies are continuing to investigate and demonstrating preferential involvement of certain subsets of Tregs in particular diseases, a major hindrance still exists due to a lack of convenient and definitive biomarkers that can distinguish between tTregs and pTregs.

Another major breakthrough is the ability to generate Tregs in large quantity for cell-based treatment to reestablish immunologic tolerance. A major therapeutic concern is that these Tregs are polyclonal in antigen-specificity and heterogeneous in composition of tTregs, iTregs, pTregs, and non-Tregs. The capability to identify and purify a more homogenous Treg population would provide a better cellular product with the potential for greater efficacious and minimal side effects. While more clinical trials are needed to translate the promising results of preclinical studies, the theoretical concerns discussed above should be taken seriously and our approach should have safe-guard mechanisms to disable their functions in the event that they become pathologic. Of men and mice are not always the same and translatable. There are still major concerns as to whether iTregs can be generated in humans. Stability and function *in vitro* or *in vivo* of humanized murine models are not equivocal to the remaining lifespan of a human being after the cells have been infused. The question is whether trading cancer for autoimmunity or exchanging one autoimmunity or another is acceptable. Nevertheless, we are encouraged and excited because of the curative potential of these novel cell-based therapies over our existing drug-based treatments. The thought of a one-time treatment to cure a condition over a lifelong administration of drugs to only prolong the inevitability of a disease is driving our innovation to achieve this development.

## Conflict of Interest Statement

The authors declare that the research was conducted in the absence of any commercial or financial relationships that could be construed as a potential conflict of interest.
